# Non-Coding Ribonucleic Acids as Diagnostic and Therapeutic Targets in Cardiac Fibrosis

**DOI:** 10.1007/s11897-024-00653-1

**Published:** 2024-03-15

**Authors:** Samuel R. Olson, W. H. Wilson Tang, Chia-Feng Liu

**Affiliations:** 1 Medicine Institute, Cleveland Clinic, Cleveland, OH 44195 USA; 2 Cardiovascular and Metabolic Sciences, Lerner Research Institute, Cleveland Clinic, 9500 Euclid Avenue, Cleveland, OH 44195 USA; 3 Kaufman Center for Heart Failure Treatment and Recovery, Heart Vascular and Thoracic Institute, Cleveland Clinic, Cleveland, OH 44195 USA

**Keywords:** Cardiac fibrosis, Epigenetic modulation, Non-coding RNAs, microRNA (miRNA), Long non-coding RNA (lncRNA), Circular RNA (circRNA), Regulatory networks

## Abstract

**Purpose of Review:**

Cardiac fibrosis is a crucial juncture following cardiac injury and a precursor for many clinical heart disease manifestations. Epigenetic modulators, particularly non-coding RNAs (ncRNAs), are gaining prominence as diagnostic and therapeutic tools.

**Recent Findings:**

miRNAs are short linear RNA molecules involved in post-transcriptional regulation; lncRNAs and circRNAs are RNA sequences greater than 200 nucleotides that also play roles in regulating gene expression through a variety of mechanisms including miRNA sponging, direct interaction with mRNA, providing protein scaffolding, and encoding their own products. NcRNAs have the capacity to regulate one another and form sophisticated regulatory networks. The individual roles and disease relevance of miRNAs, lncRNAs, and circRNAs to cardiac fibrosis have been increasingly well described, though the complexity of their interrelationships, regulatory dynamics, and context-specific roles needs further elucidation.

**Summary:**

This review provides an overview of select ncRNAs relevant in cardiac fibrosis as a surrogate for many cardiac disease states with a focus on crosstalk and regulatory networks, variable actions among different disease states, and the clinical implications thereof. Further, the clinical feasibility of diagnostic and therapeutic applications as well as the strategies underway to advance ncRNA theranostics is explored.

## Introduction

Following cardiac insult, fibrosis develops by excessive extracellular matrix (ECM) accumulation that creates tissue microenvironments affected by inflammation and oxidative stress, enhancing profibrotic signaling cascades responsible for tissue disruption and clinically detectable pathologic derangements [1, 2]. Fibrosis is an important adaptive process, but excessive fibrosis leads to organ dysfunction such as that seen in heart failure [3]. Thus, there is a strong impetus for the development of clinical tools to address fibrosis. Heart disease remains the most common cause of death in the United States, with deaths from heart disease rising from 596,577 in 2011 to 696,962 in 2020 [4]. Therapies targeting fibrosis have shown potential in preclinical research; however, many challenges remain in the translation of this work to patient care such as drug delivery and off-target effects [5].

Epigenetic regulators, which traditionally modify the expression of DNA and RNA without altering the original nucleotide sequence, have emerged as novel targets for the treatment of cardiac fibrosis and heart failure (HF) [6, 7]. Non-coding RNAs (ncRNAs) include microRNAs (miRNAs), long non-coding RNAs (lncRNAs), and circular RNAs (circRNAs), each with specific and nuanced regulatory roles [8]. MiRNAs, small 20–24 nucleotide sequences, regulate genes post-transcriptionally, typically by repressing the translation of messenger RNAs (mRNAs) [9]. MiRNA dysregulation is seen in a myriad of cardiovascular diseases, making them valuable as diagnostic and therapeutic targets [10]. LncRNAs are 200 or more nucleotide RNAs serving as key regulators of gene expression in cardiac fibrosis [11]. There are thousands of lncRNAs encoded in the human genome with a diversity of roles including direct gene enhancement or silencing, trafficking of nuclear factors, DNA repair, protein binding, sponging miRNAs, and others [12]. Like lncRNAs, circRNAs are 200 or more nucleotide-long sequences with high regulatory versatility and a unique, highly stable closed-loop structure [13]. CircRNAs also express a broad range of regulatory capabilities, often by sponging miRNAs, but also by acting as reservoirs, binding proteins directly, recruiting proteins to specific cellular sites, encoding micropeptides, and producing daughter ncRNAs [14, 15]. Importantly, many miRNAs, lncRNAs, and circRNAs have been observed to exhibit differential regulation patterns across disease states often with promiscuous targeting of mRNA, DNA, proteins, and other ncRNAs, forming sophisticated regulatory networks with high context dependence [16]. These features of ncRNAs establish the regulatory prowess foundational to their potential in diagnostic and therapeutic applications but also present cardinal challenges for clinical development.

In this review, we will examine the signaling mediators central to cardiac fibrosis and HF and define how ncRNAs interact among these pathways while also highlighting ncRNAs as potential biomarkers and future pharmacotherapies for the evaluation and treatment of fibrotic cardiac disease.

## Micro-RNAs’ Importance to Cardiac Fibrosis

There is an expanding catalog of miRNAs differentially expressed in cardiac tissues [17]. In the context of cardiac fibrosis and HF, miRNAs are crucial regulators of transforming growth factor beta (TGFβ)-signaling networks [18]. MiRNAs may bind to mRNA targets at multiple different coding or non-coding sites to silence or induce degradation, but they can also act as promoters of translation [19]. MiRNAs may be conceptualized as primary or direct epigenomic effectors themselves, regulated by secondary epitranscriptomic regulators including lncRNAs and circRNAs. However, miRNAs can also act to regulate target lncRNAs or circRNAs. For example, miR-22 and the lncRNA metastasis-associated lung adenocarcinoma transcript 1 (MALAT1) were shown to have a reciprocal relationship in endothelial cells, where inhibition of miR-22 increased lncRNA MALAT1 levels, while inhibition of lncRNA MALAT1 increased miR-22 levels [20].

As therapeutic targets, miRNAs are attractive due to their linear, single-strand structure, making them highly druggable by specific oligonucleotide inhibitors [21]. Challenges facing miRNA therapies include the tendency for a single miRNA to have multiple targets, including mRNA, DNA, proteins, or other ncRNAs, as well as the fact that perfect miRNA-target complementarity is not functionally requisite [22]. As biomarkers, circulating miRNAs have shown potential to inform diagnosis, progression, and prognosis across various cardiac disease states, including heart failure, myocardial infarction, and myocarditis [23]. Biomarker applications face challenges given that structurally, miRNAs are vulnerable to RNAse enzymes in circulation and therefore require transport either within exosomes or bound to stabilizing proteins like argonaute 2, an endogenous component of the functional miRNA complex [24]. Further, many miRNAs are conserved across tissue types, which raises challenges for the establishment of disease specificity in some cases [25].

Altogether, confidence in miRNA clinical applications remains high, although much work remains to establish feasibility in patient care. Here, we will review the mechanisms and clinical potential of select miRNAs well-studied in cardiovascular disease.

### MiR-29

MiR-29 is a central regulator of cardiac fibrosis and HF, representing a family of three miRNAs, miR-29a, miR-29b, and miR-29c [26]. Dozens of direct and indirect targets of miR-29 have been identified in cardiac fibroblasts, most importantly those for ECM components including collagen, fibrillin, and matrix metalloproteinases involved in ECM degradation [27]. The miR-29 family are high-level regulators of TGFβ signaling relevant to all fibrotic cardiovascular diseases (Table 1).

**Table 1:** Proposed roles of miR-29 family across common cardiac disease states

Disease state	Target(s)	Effects of targeting miR-29	Ref
Myocardial Infarction	PI3K/mTOR/HIF1α/VEGF; SH2B3	Inhibition via lentiviral antimir-29 led to a favorable increase in VEGF expression, while induced overexpression of miR-29 increased fibrosis	[28, 29]
Coronary atherosclerosis	*COL1A1, COL1A2, COL3A1, COL4A1, COL5A2, FBN1, MMP2*	Inhibition via antimir-29 decreased plaque stability	[30]
Congestive heart failure	*GSK3B, ICAT/CTNNBIP1, HBP1 and GLIS2*	Inhibition of miR-29 decreased hypertrophy and fibrosis	[31]
Atrial fibrillation	*Cx43*, *PDGF-B*; *TGFβ-R1*	Overexpression via AAV-miR-29b-3p decreased fibrosis	[32, 33]
Aortic aneurysm	*COL1A1, COL2A1, COL3A1, COL5A1*, Elastin, *MMP2, MMP9*	Inhibition via antimir-29b increased fibrosis by promoting ECM expression	[34]

*PI3K* phosphoinositide 3-kinase, *mTOR* mammalian target of rapamycin, *HIF1α* hypoxia-inducible factor 1-alpha, *VEGF* vascular endothelial growth factor, *SH2B3* Src homology 2B3, *COL1A1* collagen type I alpha 1 chain, *COL1A2* collagen type I alpha 2 chain, *COL3A1* collagen type III alpha 1 chain, *COL4A1* collagen type IV alpha 1 chain, *COL5A2* collagen type V alpha 2 chain, *FBN1* fibrillin 1, *MMP2* matrix metalloproteinase 2, *GSK3B* glycogen synthase kinase 3 beta, *ICAT/CTNNBIP1* inhibitor of beta-catenin, *TCF* transcription factor, *HBP1* high mobility group AT-Hook 1, *GLIS2* GLIS family zinc finger 2, *Cx43* connexin 43, *PDGF-B* platelet-derived growth factor-B, *TGFβ-R1* transforming growth factor-beta receptor 1, *MMP9* matrix metalloproteinase 9

The apparent specificity of miR-29 for fibrosis makes it an attractive target for diagnostic and therapeutic applications. MiR-29a has been described as the only biomarker specific to fibrosis in the setting of hypertrophic cardiomyopathy [35]. Similarly, miR-29b has been shown as a viable biomarker indicating fibrosis in dilated cardiomyopathy and diabetes-associated cardiac fibrosis [36, 37].

Therapeutic applications of miR-29 have been explored in preclinical studies. In a model of chronic coronary atherosclerosis, inhibition of all miR-29 isoforms resulted in a profibrotic effect; however, this was ultimately beneficial to plaque stability and the size of atherosclerotic lesions, suggesting miR-29 therapies could benefit patients with vulnerable atherosclerotic lesions by reducing coronary events and lessening the severity of ischemic cardiomyopathy [30]. In heart failure, there have been conflicting data on the role of miR-29. One study inhibited the miR-29 family using locked nucleotide antimiR-29, showing a potential therapeutic benefit to the inhibition of miR-29 by way of reduced hypertrophy and fibrosis [38]. In contrast, a similarly modeled study targeting miR-29 via cholesterol-conjugated antimiR-29b resulted in increased fibrosis [31]. So, while miR-29 represents a promising target, the specific clinical scenarios in which a clinical benefit may be achieved need to be further defined.

### MiR-21

MiR-21 has been studied as both a biomarker and therapeutic target due to its prominent expression in multiple cardiac cell types and its importance to fibrosis and heart failure (Table 2) [39]. Numerous mechanisms have been described, primarily relating to the regulation of apoptosis and the promotion of fibrosis. After myocardial infarction, miR-21 is overexpressed in cardiomyocytes at the border zone surrounding infarcted tissue and confers a protective effect by inhibition of apoptosis which may mitigate the development of ischemic cardiomyopathy [40, 41]. In heart failure, miR-21 acts to prevent apoptosis and promotes fibrosis and hypertrophy via the TGFβ-associated Smad7 and sprouty homolog (Spry1/2) proteins [42]. Acting in a profibrotic manner in a model of hypertensive cardiomyopathy, miR-21 has been shown to mediate the activation of fibroblasts and deposition of ECM via its action upon ERK-MAPK signaling, again, through the inhibition of Spry1 [43]. MiR-21 is increased in chronic atrial fibrillation and interestingly, in a non-fibrotic role, has been shown to be involved in the control of L-type calcium channels via the regulation of subunit expressions [44].

**Table 2:** MiR-21 differential expression and role in various disease states

Disease context	Expression change and role in disease	Ref
Myocardial infarction	Early upregulation in infarct region and downregulation in border zone; impact on localized cellular response in early AMI; antifibrotic	[45]
	Time-dependent change: increased 1st and 2nd weeks, unchanged 4th week; time-dependent cardioprotective effect	[46]
Atrial fibrillation	Increased expression; correlated with atrial collagen content	[47]
	Increased expression in human atrial myocytes; profibrotic, also involved in L-type calcium channel regulation	[44]
Heart failure	Increased expression with utility in predicting rehospitalization; correlates with LVEF, BNP, and galectin-3	[48, 49]

*AMI* acute myocardial infarction, *LVEF* left ventricular ejection fraction, *BNP* B-type natriuretic peptide.

MiR-21 has achieved an accuracy (AUC) of 0.76 (CI 0.71–0.82) for the diagnosis of acute myocardial infarction, but when combined with miR-1 and miR-499, accuracy has improved to 0.89 (CI 0.85–0.94); adding high-sensitivity troponin T to this panel of miRNAs further increased diagnostic accuracy to 0.94 (CI 0.92–0.97) [50]. Additionally, miR-21 has been shown to display context-dependent expression patterns in heart failure patients. Circulating miR-21 is elevated in heart failures, with particularly high levels seen during symptomatic phases [42]. Another study showed circulating miR-21 was elevated in patients with heart failure regardless of the underlying etiology and was associated with mortality and rehospitalization [48]. While circulating miR-21 is detectable with differential expression in cardiovascular disease, miR-21 has also been described across many non-cardiac pathologies which may limit its future clinical utility [51].

MiR-21 stereotypes miRNAs in cardiovascular disease as complex and enigmatic targets. MiR-21 expression is increased in multiple processes and displays multiple regulatory targets that may lead to either protective or harmful effects on overall cardiac function, making it a complex candidate for diagnostic and therapeutic use.

### MiR-133

MiR-133, an antifibrotic regulator, is among the most abundant miRNAs expressed in the heart [52]. There are three isoforms of miR-133 (miR-133a, 133b, and 133c); miR-133a has particular importance in apoptosis, fibrosis, hypertrophy, electrical conduction, and other processes [53].

In ischemic cardiomyopathy, miR-133a is involved in both early pathogenesis and late remodeling by way of reducing cardiomyocyte apoptosis, regulating angiogenesis, inhibiting TGFβ-dependent fibrosis, and repressing inflammatory cell infiltration [54]. MiR-133a may be downregulated in the ischemic myocardium to the detriment of the surviving tissue [55]. Post-mortem tissue expression of miR-133a has been shown to be significantly lower compared to that from surviving adult hearts after myocardial infarction, suggesting that miR-133a expression levels relate to survival outcomes, and lower levels are associated with increased mortality [56]. Patients with acute myocardial infarction have detectable circulating exosomal miR-133a even before troponin T elevation [57]. Further studies have also shown a time-dependent rise in serum concentrations of miR-133a, trending similarly to troponin I in the same samples [58]. The sensitivity and specificity of miR-133a for myocardial infarction are reviewed in Table 3.

**Table 3:** Sensitivity and specificity of circulating miRNAs as biomarkers in myocardial infarction

miRNA	Sensitivity [50, 59]	Specificity [50, 59]
miR-499	0.88 (95% CI, 0.86–0.90; *P* = 0.0000), 0.84 (95% CI, 0.70–0.92)	0.87 (95% CI, 0.84–0.90; *P* = 0.0000), 0.97 (95% CI, 0.87–0.99)
miR-1	0.63 (95% CI, 0.59–0.66; *P* = 0.0000), 0.72 (95% CI, 0.61–0.81)	0.76 (95% CI, 0.71–0.80; *P* = 0.0000), 0.88 (95% CI, 0.79–0.94)
miR-133a	0.89 (95% CI, 0.83–0.94; *P* = 0.0047), 0.73 (95% CI, 0.55–0.85)	0.87 (95% CI, 0.79–0.92; *P* = 0.0262), 0.88 (95% CI, 0.74–0.95)
miR-208	0.78 (95% CI, 0.76–0.81; *P* = 0.0581), 0.83 (95% CI, 0.74–0.89)	0.88 (95% CI, 0.84–0.91; *P* = 0.0000), 0.96 (95% CI, 0.82–0.99)

*Formatted for clarity with comma-separated values where the 1st value is ref. [50] and the 2nd value is ref. [59]

As a therapy, synthetic miR-133a has shown the potential to counteract ventricular remodeling and prevent heart failure in a model of pressure overload [60]. In a model of ischemic heart disease, the administration of miR-133a decreased apoptosis [61]. Interestingly, existing medications have been shown to affect miR-133a activity; the mixed β-adrenergic antagonist carvedilol reduces caspase-dependent cardiomyocyte apoptosis related to oxidative stress by increasing miR-133a expression [62]. Ivabradine, an inhibitor of sinoatrial pacemaker activity used in heart failure has also been shown to increase expression of miR-133a [63].

MiR-29, miR-21, and miR-133a do well to show both the high potential of miRNAs as drug targets and biomarkers, demonstrating the potential to detect and inhibit pathologic fibrosis at the very first steps. However, the lack of target and disease specificity is equally well-exemplified.

## Long Non-Coding RNAs in Cardiac Disease and Fibrosis

LncRNAs commonly act to sponge miRNAs for direct repression but also recruit proteins to cellular sites of interest, regulate mRNA splicing, control protein translation, express micropeptides, and form microcompartments to concentrate effector proteins [64, 65]. Scaffolding is one notable role; the lncRNA ANRIL antisense noncoding RNA in the INK4 locus attaches to its target gene and as a scaffold for the WDR5 (WD repeat-containing protein 5) and HDAC3 (histone deacetylase 3) proteins, leading to phenotype switching in vascular smooth muscle cells in a model of atherosclerotic heart disease [66]. Encoding micropeptides is another interesting function of lncRNAs; myoregulin, endoregulin, and another-regulin are lncRNA-encoded peptides that function as negative regulators of the sarcoplasmic reticulum Ca^2+^-ATPase (SERCA) critical for contractile function in the heart [67]. Like the other ncRNA classes, certain lncRNAs are differentially expressed in cardiac tissues such as lncRNA MIAT, lncRNA H19, and lncRNA MALAT1.

### LncRNA MALAT1 (Metastasis-Associated Lung Adenocarcinoma Transcript 1)

LncRNA MALAT1 has been described across a variety of cancers and cardiovascular disease states as a high-level mediator of cell migration, fibrosis, and inflammation (Table 4) [68]. After myocardial infarction (MI), lncRNA MALAT1 sponges miR-26b, restoring mitochondrial-dependent apoptosis and ultimately attenuating the progression of ischemic cardiomyopathy [69]. A tRNA-like product encoded by lncRNA MALAT1 has been described, named MALAT1-associated small cytoplasmic RNA (mascRNA). MascRNA has important immunoregulatory roles in cardiac tissue via actions upon monocyte-macrophage function, notably working in ways that are separate from its parent lncRNA MALAT1 transcripts, such as modulating expression of the Fas ligand, tumor necrosis factor-alpha (TNF-α), and interleukin-6 (IL-6) [70].

**Table 4:** Selected LncRNAs and circRNAs and their roles in cardiac pathologies

ncRNA	Model	Role	Target(s)	Effect	Ref
circRNA HIPK3	Fetal mouse cardiomyocytes	Inhibits proliferation	N1ICD, miR-133a	Stabilizes N1ICD, promotes CTGF expression	[71]
	Human cardiomyocytes, ischemia–reperfusion	Profibrotic	miR-124-3p	Upregulates Bax and downregulates Bcl-2, increasing apoptosis	[72]
	Cardiac fibroblasts, Ang-II-induced fibrosis	Profibrotic	miR-29b-3p	Upregulates a-SMA, COL1A1, COL3A1	[73]
	Cardiac fibroblasts, hypoxia	Profibrotic	miR-152-3p	Upregulates TGFβ2	[74]
circRNA Slc8a1	Pressure-overload-induced cardiac hypertrophy, neonatal mouse cardiomyocytes	Profibrotic	miR-133a	Upregulates SRF, CTGF, Adrb1, Adcy6	[75]
	Pressure overload, mouse	Cardioprotective	Unknown	Increases mitochondrial ATP synthesis	[76•]
circRNA ACTA2	Vascular smooth muscle cells	Inhibits hyperplasia and inflammation	p50 subunit of NF-κB	Downregulates NLRP3, ASC, caspase 1	[77]
lncRNA MALAT1	Myocardial infarction, mouse	Promotes apoptosis	miR-26b-5p		[69]
	Diabetic cardiomyopathy, mouse	Profibrotic	miR-141	Downregulates NLRP3	[78]
lncRNA MIAT	Angiotensin-II-induced cardiac hypertrophy	Profibrotic	miR-150	Upregulates TGFβ	[79]
	Hypertrophic cardiomyopathy	Profibrotic	miR-29a-3p	Disinhibits TGFβ-driven fibrosis	[80]
	Diabetic cardiomyopathy	Profibrotic	miR-214-3p	Upregulates IL-17	[81]
lncRNA H19	Hypoxia	Profibrotic	YB-1	Upregulates COL1A1	[82•]
		Profibrotic	miR-29a-3p/miR-29b-3p	Upregulates TGFβ2	[83]

*N1ICD* Notch1 intracellular domain, *EZH2* enhancer of zeste homolog 2, *Ang-II* angiotensin-II, *a-SMA* alpha-smooth muscle actin, *COL1A1* collagen 1A1 gene, *COL3A1* collagen 3A1 gene, *SRF* serum response factor, *CTGF* connective tissue growth factor, *Adrb1* adrenoceptor beta 1 *Adcy6* adenylate cyclase 6, *IGF-1* insulin-like growth factor 1, *IL-17* interleukin 17, *PDCD4* programmed cell death 4

Exemplifying the reciprocal relationship between miRNAs and lncRNAs, miR-144 has been shown to directly target lncRNA MALAT1 in the setting of MI, reducing its expression and thereby inhibiting apoptosis [84]. Reducing lncRNA MALAT1 expression may improve myocardial ischemia–reperfusion injury [85]. Known biomolecules have been hypothesized to affect lncRNA MALAT1; for example, the pineal gland hormone and common sleep aid melatonin were shown to have an antifibrotic effect in a model of diabetic cardiomyopathy by inhibiting lncRNA MALAT1 and miR-141-dependent NLRP3 inflammasome activation [78].

During intervention for acute MI, lncRNA MALAT1 was shown to be an effective biomarker for successful coronary “reflow” to reestablish myocardial perfusion, versus unsuccessful “no-reflow” in patients undergoing left heart catheterization; lncRNA MALAT1 expression was markedly increased in “no-reflow” patients [86•]. Circulating serum lncRNA MALAT1 performed poorly in detecting acute MI compared to other ncRNAs; it did, however, positively correlate with inflammatory cytokine levels including TNF‐α, IL‐6, and IL‐17A reiterating its importance as an inflammatory mediator in early ischemic heart disease [87].

### LncRNA MIAT (Myocardial Infarction–Associated Transcript)

LncRNA MIAT is a profibrotic regulator in the context of ischemic, hypertrophic, and other cardiomyopathies [88]. After MI, lncRNA MIAT has been shown to sponge miR-24 resulting in disinhibition of a Furin-TGFβ1 signaling cascade leading to fibrosis; when lncRNA MIAT was inhibited by a small inhibitory RNA (siRNA), cardiac function improved, supporting lncRNA MIAT as a potential drug target [89]. In another model of angiotensin-II-induced cardiac hypertrophy, targeting lncRNA MIAT with a siRNA also showed a potential to attenuate disease development by preventing lncRNA MIAT sponging of miR-150 [79].

As a biomarker, lncRNA MIAT positively correlates with acute myocardial injury and interestingly has been shown to be relatively depressed in patients with signs of transmural myocardial infarction compared to non-transmural infarction represented by the absence of ST segment elevations on electrocardiography (NSTEMI) [90]. LncRNA MIAT was proposed as an independent predictor for the development of ischemic cardiomyopathy with systolic dysfunction in the same study. Additionally, lncRNA MIAT displayed lower expression levels in patients with hypertrophic cardiomyopathy (HCM) expressing a fibrotic phenotype versus those with HCM without a fibrotic phenotype, suggesting that lncRNA MIAT may be a useful biomarker to determine the onset of fibrosis in HCM [80].

### LncRNA NEAT1 (Nuclear Paraspeckle Assembly Transcript 1)

LncRNA NEAT1 regulates inflammatory signaling cascades in cardiac fibroblasts often through the facilitation of protein–protein interactions [91]. In heart failure, lncRNA NEAT1 was found to provide scaffolding between the enhancer of zeste homolog 2 (EZH2) and the Smad7 promotor region similar to lncRNA MALAT1, thereby decreasing the expression of Smad7 and leading to unbalanced TGFβ–driven fibrosis [92]. LncRNA NEAT1 also acts traditionally via the sponging of miRNAs. Sponging miR-144 in sepsis-induced myocardial injury promoted inflammation and apoptosis [93]. Sponging miR-19a in a pressure-overload-model-induced cardiac hypertrophy [94]. Interestingly, extracellular vesicles with lncRNA NEAT1 have been shown to be involved in crosstalk between cardiomyocytes and cardiac fibroblasts in response to hypoxia; where, in this setting, lncRNA NEAT1 is silenced thereby inducing caspase-mediated apoptosis in cardiac fibroblasts [95]. These mechanisms support the potential for future lncRNA NEAT1-related therapies in HF. Finally, in one study evaluating lncRNA NEAT1 as a biomarker for heart failure, serum lncRNA NEAT1 was diagnostic, and low circulating levels were able to predict overall survival [96].

LncRNAs have a wide repertoire of regulatory functions extending beyond those seen with miRNAs. Therapeutic applications for lncRNAs offer the potential to influence many pathologic junctures in sophisticated disease states. LncRNA biomarkers may allow more specific and detailed insights. Like miRNAs, identifying the unique scenarios in which lncRNAs might be utilized is pivotal.

## Circular RNAs in Cardiac Disease and Fibrosis

The unique attribute of circRNAs is a continuous closed-loop structure, allowing for circulatory stability with resistance to nucleases that degrade linear RNAs [97, 98]. CircRNAs function as sponges to miRNAs, bind proteins directly, and like lncRNAs, can encode their own proteins [99]. CircRNAs can contain 200–2000 base pairs, giving each a massive array of potential regulatory functions with many potential targets and binding sites [100, 101]. The relationship between circRNAs and other ncRNAs has been predominantly described in terms of miRNA inhibition by sponging; however, both positive and negative regulatory mechanisms have been displayed. For example, circRNA Cdr1as, also termed ciRS-7 (circular RNA sponge for miR-7), contains many identical binding sites for miR-7, making it a potent sponge but also enabling action as a storage reservoir for miR-7 [102•]. This reservoir function has been observed in the context of ischemic cerebrovascular disease, where forced upregulation of circRNA Cdr1as resulted in the binding between circCdr1as-miR-7 with a subsequent increase in the activity of miR-7, implying that circCdr1as was acting in a protective manner toward miR-7 resulting in neuroprotection after stroke. Alternatively, in the setting of MI, circRNA Cdr1as has been shown to simultaneously sponge miR-7 and directly target its transcription factor SP1 (specificity protein 1) to promote apoptosis and increase infarct size [103].

CircRNAs are large ncRNA regulators with unique architecture and regulatory functions; their expression can be tissue- and species-specific, and taken together, these traits have garnered strong interest in the research and development of circRNA-based therapeutic and diagnostic tools. In Table 4, circRNA functions in disease development are reviewed, and in Table 5 proposed circRNA biomarkers are listed.

**Table 5:** Circulating RNA proposed as biomarkers in cardiac disease

Disease state	circRNA	Expression	Ref
Acute myocardial infarction	circRNA 104761	Downregulated	[104]
	circRNA Slc8a1	Upregulated	[105]
	circRNA NFIX	Downregulated	[105]
Coronary artery disease*	circRNA 0001946	Upregulated	[106]
	circRNA ZNF609	Downregulated	[107]
	circRNA 0005540	Upregulated	[108]
	circRNA NPHP4	Upregulated	[109]
	circRNA 0124644	Upregulated	[110]
	circRNA 0001879	Upregulated	[111]
	circRNA 0001445	Upregulated	[112]
	circRNA YOD1	Upregulated	[113]
Compensated heart failure*	circRNA 0062960	Upregulated	[114]
Aortic dissection (Stanford type A)*	circRNA MARK3	Upregulated	[115]
Kawasaki disease*	circRNA ANRIL	Downregulated	[116]
Atrial fibrillation*	circRNA 025016	Upregulated	[117]

*No specific biomarker exists currently for coronary artery disease, compensated heart failure, aortic dissection, Kawasaki disease, or atrial fibrillation, thereby establishing these circRNAs as first-in-class

### CircRNA Slc8a1 (Solute Carrier Family 8 Member A1)

The Slc8a1 gene encodes the circRNA Slc8a1 (circSlc8a1), highly expressed in cardiomyocytes and implicated in a variety of cardiac disease states including ischemic cardiomyopathy and pressure-overload-induced heart failure [75, 118]. CircSlc8a1 may have pro- or anti-fibrotic actions. In heart failure, circRNA Slc8a1 sponges and inhibits miR-133a, disallowing its antifibrotic actions on TGFβ signaling, specifically the regulation of serum response factor (SRF) and connective tissue growth factor (CTGF) expression as well as components of the β1-adrenergic receptor transduction cascade [76•]. Exemplifying its therapeutic potential, this study deployed a short-hairpin RNA to target and inhibit endogenous circRNA Slc8a1, which in turn resulted in the disinhibition of miR-133a and reduction of pathological hypertrophy and fibrosis in the model of pressure-overload-induced heart failure. In contrast to these proposed profibrotic functions, a study by Wu et al. [76•] showed that induced overexpression of circRNA Slc8a1 was in fact cardioprotective in a pressure-overload model via promotion of mitochondrial ATP synthesis. In their study, for the first time, an antisense circRNA was developed (cA-circSlc8a1) to silence circRNA Slc8a1, leading to severe congestive heart failure in this model. Endogenous antisense circRNAs have been described in various disease states, though the proposal of artificial antisense circRNAs in drug development has not been deeply explored and may be a future avenue for circRNA therapeutic applications [119].

### CircRNA HIPK3 (Homeodomain-Interacting Protein Kinase 3 Gene)

Like circRNA Slc8a1, circRNA HIPK3 has been shown to sponge miR-133a in a model of MI, enhancing Notch1 and connective tissue growth factor (CTGF) expression thereby driving proliferation, migration, and fibrosis in a manner protective against heart failure [120, 121]. These regulatory functions of circRNA HIPK3 involving CTGF and Notch1 have been shown to culminate in a decreased fibrotic area after MI which may attenuate the development of ischemic cardiomyopathy [71, 122]. Drugs targeting circRNA HIPK3 could be potent modulators of TGFβ-driven fibrosis and heart failure; however, its context dependence may not be completely understood. In a study of human-derived cardiomyocytes, circRNA HIPK3 expression was seen to be detrimental after stimulation of ischemia–reperfusion injury by sponging miR-124-3p, where the proapoptotic Bax (apoptosis regulator BAX) was disinhibited, and the antiapoptotic Bcl-2 (B-cell lymphoma 2) was inhibited [72]. This effect on cardiomyocyte apoptosis reiterates findings in another ischemia–reperfusion model, where miR-29a was shown to be a target of circRNA HIPK3, resulting in increased apoptosis of cultured human cardiomyocytes via the disinhibition of insulin-like growth factor 1 (IGF-1) signaling [73]. Altogether, the potential clinical benefit of targeting circRNA HIPK3 remains to be seen.

### CircACTA2 (Alpha-Smooth Muscle Actin)

CircRNA ACTA2 regulates vascular smooth muscle cells via alpha-smooth muscle actin (α-SMA), a hallmark product of activated fibroblasts functioning in cellular motility and smooth muscle contraction [123]. Dysregulation of α-SMA has been observed in multiple cardiovascular disease states including HF, coronary atherosclerosis, and stroke [124].

Similar to the lncRNA MALAT1-miR-141 network, circACTA2 has been shown to inhibit the activation of the NLRP3 inflammasome in vascular smooth muscle cells [125]. In this study, an artificial circRNA ACTA2 was able to attenuate the expression of NLRP3, ASC, and caspase 1, exposing the axis as a potential drug target in vascular disease. Chronically elevated angiotensin-II, seen in hypertension and hypertrophic cardiomyopathy, results in senescence of VSMCs mediated by circACTA2 actions upon interleukin enhancer-binding factor 3 (ILF3) [77]. CircRNA ACTA2 acts as a competing endogenous RNA against the mRNA for cyclin-dependent kinase 4 (CDK4), preventing what would be a CDK4 mRNA-ILF3 gene interaction, thereby leading to cell senescence due to obsoletion of the CDK4 mRNA. The therapeutic candidacy of circRNA ACTA2 was also demonstrated in these mechanistic studies; however, there has not yet been any investigation of circRNA ACTA2 as a biomarker for cardiac fibrosis or heart disease.

In summary, circRNAs, characterized by their closed-loop structure, exhibit unique and significant regulatory roles in heart failure and fibrosis. Notable examples include circRNA Slc8a1 modulation of miR-133a in fibrosis, circRNA HIPK3 in MI and apoptosis, and circRNA ACTA2 impact on vascular inflammation. These circRNAs demonstrate complex, context-dependent regulatory capabilities, highlighting their potential as both biomarkers and therapeutic targets in cardiac pathology.

## Clinical Diagnostic Applications of Non-Coding RNAs

Non-coding RNAs hold promise as next-generation biomarkers. With high disease-, tissue-, or cell-type dependence, ncRNAs could offer not only superior sensitivity and specificity but also use across a wide range of cardiac diseases, identifying not only the presence or absence of a disease process, but also reflecting stages in disease development, the severity of disease, and underlying tissue processes, i.e., fibrosis.

Linear single-stranded ncRNAs such as miRNAs are intrinsically vulnerable to degradation and can only exist in circulation by vesicular transit or binding to stabilizing proteins [126]. Another challenge facing the development of ncRNA biomarkers is specificity; miR-21 has been described as a biomarker for at least 29 different pathologies [51]. Further, splicing of ncRNAs creates high heterogeneity, and while this expands their potential functions, it also adds another layer of complexity to the development of clinical tools [127].

Many ncRNAs have detectable changes in expression patterns in the setting of ischemic cardiomyopathy and MI (Table 3). Current troponin-based assays represent the standard of care for the diagnosis of acute myocardial ischemia, but interest remains for biomarkers offering superior sensitivity and specificity [59, 128]. In MI, miR-137 and miR-106b-5p are released within 5 min by ischemic myocardium before the onset of necrosis offering an advantage over traditional troponin biomarker assays [129]. Some circRNAs are expressed in the chronic dormant phase of coronary atherosclerotic disease, such as circYOD1 [113]. A biomarker for chronic coronary atherosclerosis would be a significant milestone as there is no available biomarker to diagnose chronic coronary disease today and could have implications for the detection and prevention of ischemic cardiomyopathy. Utilizing the context-dependent expression of ncRNAs is also of interest in heart failures, for example, in the observation of differential expression of miR-21 during decompensated HF [48]. There are an increasing number of potential ncRNA candidates for cardiac disease states that presently lack any specific biomarker, such as heart failure with preserved ejection fraction, myocarditis, atrial fibrillation, and aortic dissection (Table 5 and 6). Altogether, the future diagnostic applications of ncRNAs are an exciting prospect in the context of cardiac disease.

**Table 6:** Novel miRNA biomarkers for orphaned cardiac disease states

Disease state	miRNA(s)	Ref
Myocarditis	miR-Chr8:96	[130]
	miR-155	[131]
	miR-206	[132]
Atrial fibrillation	miRNA-328, miRNA-223-3p, miRNA-21, miRNA-29b, miRNA-1-5p	[133]
Heart failure with reduced ejection fraction	miR-18b-3p, miR-21-5p, miR-22-3p, miR-92b-3p, miR-129-5p, miR-320a-5p, miR-423-5p, miR-675-5p	[134]
Heart failure with preserved ejection fraction	miR-19b-3p, miR-30c-5p, miR-206, miR-221-3p, miR-328-5p, miR-375-3p, miR-424-5p	[134]

## Clinical Therapeutic Applications of Non-Coding RNAs

No miRNA drugs have yet advanced to late-stage clinical development for cardiac disease, though other gene therapies have entered the market, perhaps as forerunners to miRNA and other ncRNA drugs. Like miRNAs, small interfering RNAs (siRNAs) are ncRNAs that target specific mRNA sequences [135]. Vutrisiran and patisiran are two siRNA drugs that suppress amyloid transthyretin (aTTR) mRNA in the liver, currently approved for the treatment of polyneuropathy related to hereditary aTTR amyloidosis [136, 137]. Both siRNAs have come under investigation for the treatment of aTTR cardiac amyloidosis, a rare but important cause of HF [138, 139]. Unlike the other ncRNAs, siRNAs have especially high specificity and are designed to display total complementarity to their targets. SiRNAs target a single mRNA with minimal risk of off-target binding, whereas miRNAs can be synthesized to mimic the effect of any other ncRNA with multiple mRNA or non-mRNA targets, or, in theory, to inhibit any target miRNA, lncRNA, or circRNA all while lacking perfect complementarity.

Drug delivery is a challenge with gene therapies. Studies in this review utilized various delivery vectors such as viral vectors, antagomirs, and stable oligonucleotides. Numerous strategies remain under investigation to improve delivery in vivo. Lipid nanoparticle systems can readily store and carry RNAs, and PEGylation of liposomes—conjugation of polyethylene glycol (PEG) to prevent degradation—may reduce potential toxicity and nonspecific uptake; patisiran is one example of this delivery system [140]. Exosomal delivery systems have also been of interest, as endogenous exosomal ncRNA regulatory networks have been described in vivo, supporting the hypothesis that artificial exosomes for ncRNA delivery may offer advantages to transfection efficacy, stability, and carrying capacity [141].

Unlike siRNAs, miRNAs, circRNAs, and lncRNAs have multiple targets and perform far more complex and context-dependent regulatory functions. NcRNAs may regulate any level of transcription or translation, epigenetic modification, or proteins directly. While indeed this presents a risk for unintended consequences, it also offers the potential for far more potent suppression of the complex maladaptive signaling pathways seen in cardiovascular fibrosis and heart failure, should these challenges be overcome.

## Conclusion

Cardiac fibrosis is the greatest commonality in acquired cardiovascular diseases and will eventually lead to heart failure. MiRNAs, lncRNAs, and circRNAs have emerged as pivotal regulators at nearly all levels of pathologic fibrosis, and modulating their expression has shown great potential for reversing pathologic disease states. As described, miRNAs not only target specific mRNA sequences but also regulate other ncRNAs within complex networks. LncRNAs can sponge many different RNA targets and their roles as recruiters, scaffolds for other biomolecular interactions, or protein encoders continue to emerge. CircRNAs are increasingly recognized as critical diagnostic and therapeutic targets given their stability in addition to high-level regulatory functions in cardiovascular disease, and their structure enables a huge variety of functional capabilities while also vastly increasing the regulatory potency of any given circRNA.

Many ncRNAs have emerged as circulating biomarkers in different cardiac diseases, and clinical detection of these biomarkers has become increasingly realistic. In addition to improving upon existing clinical assays, ncRNAs offer the potential to emerge as biomarkers for cardiac diseases that have none today such as heart failure with preserved ejection fraction, atrial fibrillation, or myocarditis. Further, specific ncRNAs have the potential to inform the stage of disease and the underlying cellular processes such as whether fibrosis is currently occurring in the tissue. Methods to detect these circulating ncRNAs continue to evolve to overcome challenges such as speed, availability, and labor requirements.

As therapeutics targeting cardiac fibrosis, miRNAs, lncRNAs, and circRNAs may be the next gene therapies following the budding class of siRNA drugs. Fibrosis is a sophisticated and multifactorial process in cardiac disease. We reviewed some of the intricacies of fibrogenesis among TGFβ pathways, where miRNAs, lncRNAs, and circRNAs show the ability to target many different biomolecules to inhibit profibrotic signaling at multiple stages and with potent effects. Further work is needed toward mitigating the risks of off-target effects and degradation in circulation for these ncRNAs.

Regulatory interrelationships and the contextual variance of ncRNAs have emerged as areas of paramount importance, carrying implications for both disease descriptions and therapeutic interventions. Looking forward, the potential for targeted ncRNA therapies and diagnostic tools specific for cardiac fibrosis and diseases thereof seems promising. As the nuances of ncRNA functions and regulatory interactions among fibrotic signaling continue to be unraveled, so will progress be made for their clinical development.

## Data Availability

No datasets were generated or analysed during the current study.
